# Novel Insight into the Role of the Kiss1/GPR54 System in Energy Metabolism in Major Metabolic Organs

**DOI:** 10.3390/cells11193148

**Published:** 2022-10-06

**Authors:** Xuehan Li, Chunyu Liang, Yi Yan

**Affiliations:** Sport Science College, Beijing Sport University, Beijing 100084, China

**Keywords:** Kiss1/GPR54 system, kisspeptin, energy metabolism

## Abstract

The Kiss1/GPR54 system is a multifunctional genetic system with an essential role in regulating energy balance and metabolic homeostasis. In the mammalian hypothalamus, two major populations of neurons, the rostral periventricular region of the third ventricle (RP3V) and the arcuate nucleus (ARC), produced kisspeptin. Kiss1^ARC^ neurons input kisspeptin and glutamate to feeding-associated neurons to regulate energy intake and expenditure balance. Kisspeptin in the peripheral circulation is involved in lipid accumulation in adipose tissue. In the hepatic and pancreatic circuits, kisspeptin signaling affects insulin secretion, suggesting the critical role of the Kiss1/GPR54 system in regulating glucose and lipid metabolism. In addition, this review also predicts the role of the Kiss1/GPRS4 system in skeletal muscle in association with exercise performance. Recent studies have focused on the link between kisspeptin signaling and energy homeostasis, further investigation of potential function is warranted. Therefore, this review summarizes the role of the Kiss1/GPRS4 system in the major metabolic organs in relation to energy metabolism homeostasis, aiming to endow the reader with a critical and updated view of the Kiss1/GPR54 system in energy metabolism.

## 1. Introduction

**Figure 1:**
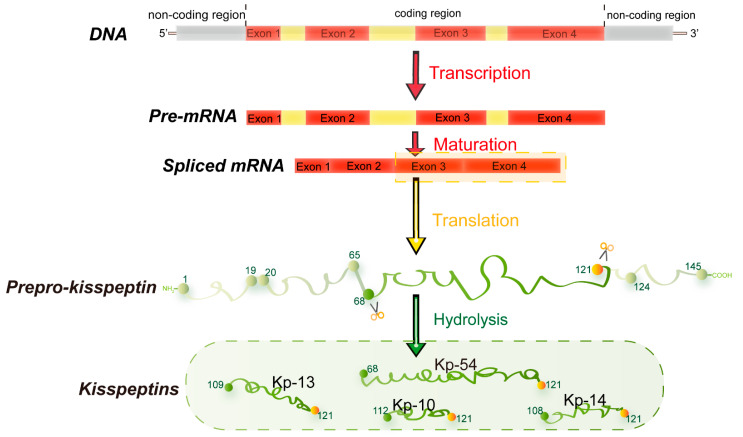
Major structural features of human kisspeptin gene. The human *Kiss1* gene consists of four exons, the last two encoding a precursor protein translated into a 145 amino acid. This precursor protein includes a predicted signal peptide of amino acids 1-19, a potential dibasic cleavage site of amino acids 65-68, and a terminal cleavage and amidation site of amino acids 121-124. It can be fragmented into different lengths of kisspeptin with a common amidated C-terminal (KP-54/14/13/10) [7].

In 1996, metastin, encoded by the *Kiss1* gene, was found to have the ability to suppress metastatic decrease in melanoma cells [1,2]. Since then, metastin has attracted considerable attention as a metastasis suppressor and was subsequently named kisspeptin. *Kiss1*, located at the human chromosome 1q32, was identified through subtractive hybridization [3]. The encoded common precursor protein (kisspeptin) contains 145 amino acids, which can be hydrolyzed to generate multiple endogenous mature peptides with a common amidated C-terminal [4], including kisspeptin-54/14/13/10 (Kp-54/14/13/10) (Figure 1) [5]. Kp-10 is the smallest peptide that can activate its receptor and function [6]. Therefore, Kp-10 is considered the predominant form of kisspeptin.

In 2001, researchers used three independent experiments to confirm the existence of a unique receptor of kisspeptin and named it hOT7T175 or AXOR12 [8,9]. As a G protein-coupled receptor, this receptor is found in human chromosome group 19p13.3 and intracellularly coupled to the Gαq/11 subfamily of Gq/11G proteins in cells [10]. Thus, it was eventually renamed GPR54 or Kiss1r. GPR54 consists of five exons and encodes a 398 (395 in mice and 396 in rats) amino acid protein in humans [6]. The activation of GPR54 by kisspeptin causes the activation of Gαq/intracellular Ca^2+^, mitogen-activated protein kinase, and phosphatidylinositol 3-kinase/AKT pathways [11,12]. In previous studies, the Kiss1/GPRS4 system has been shown to play an important role in mammalian reproductive function and cancer biology.

*Kiss1* and *GPR54* are widely expressed in the hypothalamus, some peripheral tissues, including adipose tissues [13], and the liver [14], stomach, and pancreas [15,16]. Kisspeptin signaling functions as an anorexia factor, reproductive hormone, behavioral hormone, or transfer inhibitor in different tissues [17,18,19]. Hypothalamic Kiss1 neurons are the regulatory centers of reproductive function and the active molecules of energy balance in the central circuit [20]. Peripheral kisspeptin is mainly secreted by endocrine organs, such as adipose tissues and the liver. They are essential peptides that regulate lipid accumulation and fatty acid metabolism and contribute to glucose homeostasis [21,22]. This review summarizes current evidence showing that kisspeptin plays a role in regulating energy homeostasis by modulating multi-organ function in animals and humans and discusses the controversies within the field of the Kiss1/GPR54 system and the potential physiological implications of the Kiss1/GPR54 system.

## 2. Hypothalamic Kiss1/GPR54 System

Puberty is defined as a complex biological process involving gonadal development. Accumulating evidence suggests that hypothalamic kisspeptin is a crucial factor in the initiation of puberty. The hypothalamic–pituitary–gonadal axis is the critical endocrine system for the development of the mammalian reproductive system, especially when puberty begins [23]. In the rodent hypothalamus, Kiss1 neurons are mainly distributed in two locations: arcuate nucleus (ARC) and the rostral periventricular region of the third ventricle (RP3V), projecting mainly to the anteroventral periventricular nucleus (AVPV) [24]. Kisspeptin secreted by Kiss1^ARC^ neurons stimulates GnRH/gonadotropin pulses that induce the secretion of pituitary gonadotropins and consequently stimulate pubertal development [25,26]. Moreover, the number of kisspeptin neurons in female mouse AVPV increased sevenfold during puberty [27]. During mammalian puberty, the methylation of the silent polycomb group gene of the hypothalamic *Kiss1* increases, activating the *Kiss1* promoter [28,29]. *Kiss1* gene expression increases 1.61-fold (1.14/0.71) in the hypothalamus of pubertal mice compared with prepubertal mice. By contrast, the growth data for GnRH and luteinizing hormone (LH) gene expression are 1.98-fold (1.07/0.54) and 38.21-fold (14.90/0.39) [30]. Diseases manifesting gonadal dysplasia, such as idiopathic hypogonadotropic hypogonadism [31] and polycystic ovarian syndrome [32], are associated with disruption to energy balance. These disorders are inextricably linked to kisspeptin.

### 2.1. Hypothalamic Kiss1/GPR54 System Regulates Pubertal Initiation by Regulating Food Intake

The onset of puberty requires sufficient energy accumulation, and in the event of a negative energy balance (e.g., fasting, anorexia nervosa, or excessive exercise), puberty initiation is delayed or inhibited [33]. Food intake is the primary form of energy accumulation in mammals. The hypothalamic Kiss1/GPR54 system can sense an organism’s energy state and regulate puberty onset by regulating food intake. Administering kisspeptin intracerebroventricular to immature and malnourished female rats restores vaginal opening and induces gonadotropin and estrogen secretion in approximately 60% of the female rats [34,35]. Furthermore, the intracerebroventricular infusion of kisspeptin-10 to chronically malnourished rats increases ovarian weight and LH levels in plasma [35]. Peripheral and central kisspeptin injections can promote sexual maturation in mammals with negative energy balance.

**Figure 2:**
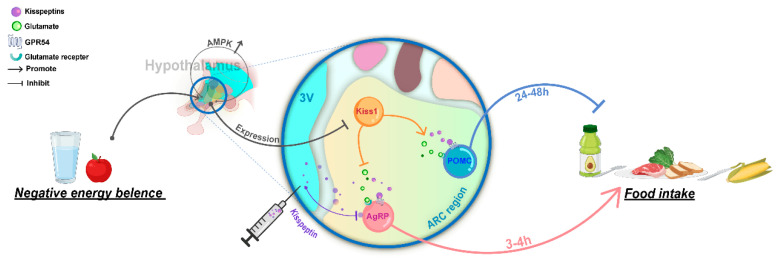
Hypothalamic Kiss1/GPR54 system regulation on food intake. Negative energy balance activates hypothalamic *AMPK*, which decreases *Kiss1* expression. The Kiss1 neurons control food intake in the ARC region via signaling molecules AGRP and POMC neurons, improving the negative energy status. In addition, injection of exogenous kisspeptin reduces food intake by inhibiting AGRP neurons.

AMP-activated protein kinase (AMPK) is a fundamental cellular energy gauge [36]. When energy is insufficient, AMPK is activated to regulate whole-body energy balance [37]. AMPK in hypothalamic neurons is activated and reduces Kiss1 expression in a mice model of chronic malnutrition (continuous caloric restriction of 30%), but the mice with conditional ablation of AMPK in Kiss1 neurons did not display overt differences in relevant indices of puberty onset [38]. Does this effect indicate a negative correlation between reduced hypothalamic *Kiss1* expression of kisspeptin and food intake? Evidence shows that appetite is suppressed in mice within 4 h after the intraperitoneal injection of kisspeptin [39]. Similarly, the central injection of kisspeptin significantly reduces the amount of food eaten by male rats within 3 h [40]. The feeding-associated neurons proopiomelanocortin (POMC) and agouti-related peptide (AgRP)/neuropeptide Y (NPY) neurons are the primary projections of Kiss1^ARC^ neurons [28,41]. POMC neurons produce peptides, including the alpha-melanocyte-stimulating hormone, which can promote satiety and reduce food intake while increasing energy expenditure. Conversely, AgRP and NPY peptides produced by AgRP neurons can increase food intake and decrease energy expenditure [42]. Notably, POMC and AgRP neurons can express *GPR54* [26], and kisspeptin increases the expression of *Pomc* in the ARC [43]. However, the satiety-inducing effects of POMC neuron activation are incredibly slow, typically taking 24–48 h to suppress food intake [44]. Thus, the appetite-suppressing effect of exogenous kisspeptin may be mediated by AgRP neurons, which exert the main action, rather than by POMC neurons. Then, glutamatergic neurons, with glutamate as the neurotransmitter, constitute the majority of excitatory neurons and occupy a major part of the cerebral cortex [45]. As multifunctional neurons, Kiss1^ARC^ neurons not only input kisspeptin but also glutamate to POMC and AgRP neurons. By the activation of distinct metabotropic glutamate receptors, glutamate mediates the excitation of Kiss1^ARC^ neurons on POMC neurons and selectively inhibits AgRP neurons [46,47], causing the eventual inhibition of food intake (Figure 2).

However, negative energy balance not only delays the initiation of puberty but also disrupts energy metabolism. Exogenous kisspeptin can restore partial sexual maturation in mice with negative energy balance, but metabolic disturbances persist. By contrast, Kiss1^ARC^ neurons influence feeding and physical activity via POMC and AgRP neurons, providing a buffer state to adjust to the failure of puberty initiation in mammals under the condition of negative energy balance.

### 2.2. Hypothalamic KISS1/GPR54 System Mediates the Effect of Peripheral Energy Homeostasis Regulatory Hormones on Pubertal Initiation

Some peripheral hormones, such as insulin, leptin, glucagon-like peptide-1 (GLP-1), and ghrelin, are involved in energy homeostasis regulation, cross the blood–brain barrier, and stimulate Kiss1^ARC^ neurons to secrete kisspeptin. Male and female mice lacking insulin receptors in Kiss1 neurons exhibit delayed sexual maturation or reduced LH secretion [48]. However, the deletion of insulin receptors in Kiss1 neurons does not alter LH levels in adult lean mice [49]. This finding indicates that hypothalamic kisspeptin plays a crucial role in the pubertal process and is closely related to insulin signaling. Leptin is a significant regulator in hypothalamic *Kiss1* expression. Approximately 40% of Kiss1 neurons in the ARC of male mice have functional leptin receptors (LepR) [50]. This functional receptor is not expressed before puberty; when puberty is reached, 8–15% of ARC Kiss1 neurons co-express LepR [51]. Interestingly, the central nervous system of ob/ob mice does not express leptin receptors in a physiological state. However, the administration of central leptin to pubescent female mice decreased the expression levels of *Kiss1*, *GnRH*, and *LH* after 4 h [30]. That means even in the absence of LepR, leptin can still associate with Kiss1/GPR54 system via a compensatory effect and thus may regulate LH secretion. 

In addition, many common hormones are closely related to the Kiss1/GPR54 system. For instance, a study isolated mouse islets and detected a repressive effect of ghrelin at 10^−6^ M against the transcription of the mRNA of Kiss1 and GPR54 [52]. In addition, intravenous administration of ghrelin can downregulate Kiss1 mRNA in the POA region of female rats, thus indirectly inhibiting LH pulse frequency and delaying the initiation of puberty [53]. GLP-1 is also a typical ingestion-related hormone, and the GLP-1 precursor expressed in the brain is inhibited during fasting [54]. We have already mentioned that fasting leads to a state of negative energy balance in which Kiss1 neuron expression and LH pulse frequency are suppressed. It was demonstrated that GLP-1 significantly increased Kiss1 mRNA expression in the rat clonal hypothalamic cell line rHypoE-8 [55], but liraglutide (a long-acting GLP-1R agonist) treatment is not sufficient to prevent LH inhibition from fasting [56]. In 2021, it was found that only about 5% of Kiss1 neurons in the hypothalamus of female sheep can express GLP-1R. Whether this insufficiently potent association is related to the amount of GLP-1R requires further study [57]. Furthermore, relaxin-3 belongs to the insulin superfamily. It is expressed in the hypothalamus and usually promotes appetite and lipid accumulation [58]. In mHypoA-55 cells differentiated from the ARC region, relaxin-3 of 1 nM significantly upregulates the expression of Kiss1 and GnRH mRNA. In GT1-7 cells, relaxin-3 significantly upregulates the expression of Kiss1 but has no significant effects on the expression levels of GnRH and mRNA in GT1-7 cells. In mHypoA-50 cells differentiated from the AVPV region, relaxin-3 cannot significantly affect Kiss1 expression. This work further illustrates that the insulin superfamily regulates energy homeostasis and reproductive balance by affecting Kiss1^ARC^ [59]. Thus, the hypothalamic Kiss1/GPR54 system may mediate the regulation of peripheral hormones on the pubertal sexual maturation, feeding, physical activity, and energy expenditure of the organism (discussed in later sections), but more research is needed.

## 3. Peripheral Kiss1/GPR54 System

As we have already mentioned, *Kiss1* and *GPR54* are equally widely expressed in peripheral tissues [13,14,15,16,17,19,60]. With an increasing understanding of the Kiss1/GPR54 system, researchers have realized that it is a linker for energy accumulation, metabolic homeostasis, and even disease healing [13,61]. Systemic GPR54 KO negatively affects the body weights of mice; characterized by decreased metabolic capacity and increased body weight [62]. Interestingly, female mice with conditional *GPR54* knockout exclusively in brown adipose tissue (BAT) are associated with enhanced basal metabolism, increased body temperature, and decreased body weight compared with systemic GPR54 KO mice [25,62]. Such a link between changes causing systemic metabolism and conditional knockout indicates that the role of the Kiss1/GPR54 system in adipose tissue may be related to the accumulation of lipids.

### 3.1. Kiss1/GPR54 System Involved in Lipid Accumulation

In peripheral tissues, the Kiss1/GPR54 system in adipose tissues has the highest expression level [60]. The role of the Kiss1/GPR54 system in adipose tissues is directed toward energy metabolism. The intraventricular injection of Kp-10 resulted in a significant decrease in the body weights of mice; the overall body weight of the mice in the control group was higher than that in the Kp-10-injected group after the injection of the kisspeptin antagonist p234; in the p234 group, the percentage of linoleic acid in the adipose tissues of mice was higher than that of the control group; these results indicated that the catabolism of linoleic acid in mice is compromised when kisspeptin signaling in the hypothalamus is inhibited [63]. A strong negative correlation exists between plasma triglyceride concentrations and the number of Kiss1^ARC^ neurons in rats [64]. All these results suggested that kisspeptin signaling plays a role in the metabolic regulation of fatty acids by relevant receptors in peripheral tissues.

**Figure 3:**
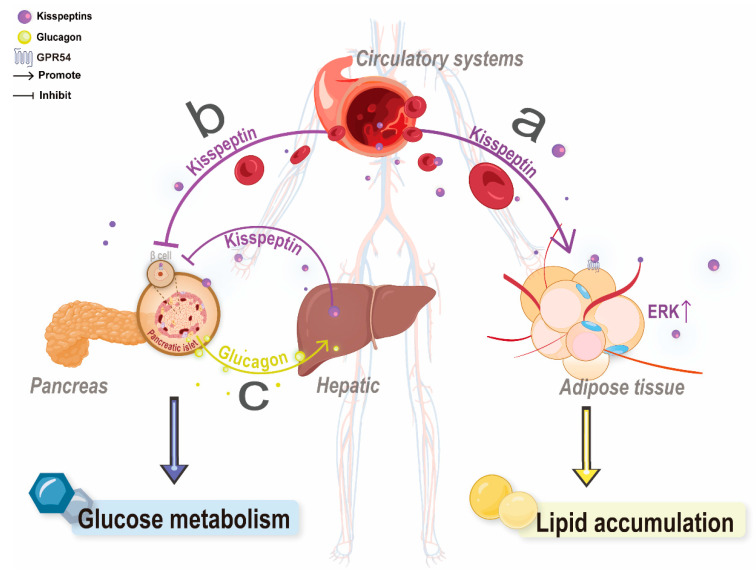
Peripheral kisspeptin regulation of glucose and lipid metabolism. (**a**) Circulating kisspeptin promotes lipid accumulation in adipose tissue. (**b**) Circulating kisspeptin stimulates pancreatic β-cells to reduce insulin secretion. (**c**) Hepatic secretion of kisspeptin stimulates pancreatic beta cells and impairs insulin secretion, while glucagon promotes hepatic secretion of kisspeptin and deteriorates glucose homeostasis (hepatic–pancreatic circuit).

In addition, the kisspeptin concentrations of prepubertal obese girls were higher than those in healthy girls, whereas healthy adult females had higher kisspeptin concentrations than immature girls, suggesting that the dosage of kisspeptin in the plasma is critical to the accumulation of adipose tissues [65]. This phenomenon is supported by additional evidence that Kp-10 at the 1 nM level promotes triglyceride synthesis in 3T3-L1 cells while accelerating the differentiation of 3T3-L1 to adipocytes. Moreover, the phosphorylation levels of extracellular regulated protein kinases (ERK) involved in the GPR54 signaling pathway were significantly reduced in the adipose tissues of castrated or ovariectomized GPR54 KO mice; this result suggested that kisspeptin promotes lipid synthesis and obesity development by activating GPR54 and then the MAP kinase pathway, as ERK phosphorylation level may be a landmark of adipocyte proliferation [66] (Figure 3). These results may explain the accumulation of adipose tissues in prepubertal obese girls. Kp-10 treats 3T3-L1 cells and inhibits adipocyte synthesis and differentiation, which may arise due to toxicity caused by a high Kp-10 concentration that leads to the abnormal state of the cells [67]. However, these results still need to be verified. Even so, they revealed the role of kisspeptin signaling in the mammalian lipogenic system, suggesting that kisspeptin signaling homeostasis is closely related to lipid metabolism.

Currently, studies on the Kiss1/GPR54 system in adipose tissue have involved the knockout of GPR54 in adipose tissue to obstruct adipose tissue from receiving kisspeptin signaling [18,25,68]. However, as the largest endocrine organ [61], kisspeptin signaling secreted by adipose tissue per se is also the key for investigation. Therefore, future studies on the Kiss1/GPR54 system in adipose tissues should concentrate on the effect of kisspeptin secreted by adipose tissues on energy homeostasis.

### 3.2. Kiss1/GPR54 System in the Hepato-Pancreatic Circuitry

The Kiss1/GPR54 system in the pancreas and hepatic system can affect blood glucose concentrations in diabetic model animals. Kisspeptin levels in the pancreas were significantly downregulated in rats with diet-induced obesity and type 2 diabetes mellitus (T2DM). At increased glucose concentrations (T1DM and T2DM rats), the Kiss1/GPR54 system cannot control insulin secretion [69]. In diabetic model mice, including those with high-fat-induced obesity and genotypic obesity (db/db and ob/ob type mice), *Kiss1* expression in the liver and the level of circulating kisspeptin increased [21,70,71]. The onset of T2DM is often accompanied by impaired glucose-stimulated insulin secretion (GSIS), which usually occurs early in the course of the disease [72]. Glucagon in mice stimulates the production of kisspeptin in the liver through cAMP–PKA–CREB signaling and binds to GPR54 on insulin β cells via the blood, thereby inhibiting islet cAMP synthesis and suppressing GSIS [21] (Figure 3). Thus, the elevation of kisspeptin level in the plasma might also be a possible new way to determine T2DM.

In healthy humans or mice, kisspeptin is usually at picomolar or nanomolar levels and essential for maintaining the relative stability of glucose, insulin, and GSIS [73,74]. Interestingly, kisspeptin at nanomolar concentrations inhibits GSIS in normal mouse islets but not in islets lacking GPR54; by contrast, kisspeptin at micromolar concentrations enhanced GSIS in normal mouse islets even in the absence of GPR54 [21]. This result suggested that kisspeptin with a level above the normal range (micromolar concentrations) may bind to other receptors and thus stimulate insulin secretion in pancreatic β-cells. However, kisspeptin at abnormally high concentrations does not function as a hormone. In other words, within a normal range (not higher than nmol levels), high levels of circulating kisspeptin indicate disrupted glucose metabolism. This phenomenon has prompted us to consider the role of circulating kisspeptin in energy metabolism, including the aforementioned impaired energy metabolic rate in global GPR54 KO rats and the relatively increased efficiency of GPR54 energy metabolism in conditional KO BAT [60].

We propose that the failure of kisspeptin to bind to GPR54 at established sites in the peripheral circulation will lead to the accumulation of circulating kisspeptin with nowhere to depart, which may in turn trigger alterations in energy metabolism by proteins other than GPR54. This hypothesis provides a new perspective on the function of the kiss1/GPR54 system in glucose and lipid metabolism, which still needs further investigation.

## 4. Possible Role of Kiss1/GPR54 System in Skeletal Muscle

GPR54 exists in human vascular smooth muscles [75,76], skeletal muscles of frogs [77], and skeletal muscles of mice [60], and Kiss1 is expressed in Rohu’s skeletal muscles [78]. In the cardiovascular smooth muscles of humans, kisspeptin can induce inotropic actions on cardiac function [76]. The Kiss1/GPR54 system in skeletal muscles has not been thoroughly studied. Only partial evidence in the skeletal muscle of some animals is available. The mRNA expression levels of Kiss1 and GPR54 are lower in mouse skeletal muscles than those in adipose tissues and the liver. However, whether a small amount of GPR54 in skeletal muscles can react with kisspeptin in the plasma is still unknown. More studies are needed to confirm the intrinsic link between Kiss1/GPR54 system and skeletal muscles.

The skeletal muscle is the storage organ for muscle glycogen and the most prominent motor organ of an organism. Consequently, the relationship between the skeletal muscle and energy metabolism cannot be ignored during physical activities, especially during exercise [79]. Although no exact relationship between the Kiss1/GPR54 system and energy balance in the skeletal muscle has been confirmed, understanding the intracellular pathways of Kiss1 with regard to energy metabolism and the possible role of the intracellular Kiss1/GPR54 system during exercise is important.

### 4.1. Kiss1/GPR54 System Affects Ca^2+^ Signaling in Cells

In the skeletal muscle, Ca^2+^ is a key signaling molecule for proliferation and differentiation [80,81,82]. The proliferation of muscle cells requires the proliferation of mesodermal stem cells, which then gradually specialize into myogenic progenitors and further differentiate into different types of muscle cells [83]. The in vitro differentiation of myoblasts is regulated by an increase in intracellular Ca^2+^ induced by changes in membrane potential [84,85]. In RyR1 homozygous mutant mice, RyR-mediated Ca^2+^ release is eliminated, and then perinatal death and severe musculature disorders, including small myotubes and disorganized myofibrils, occur [86]. In addition, the moderate-intensity-exercise-induced adaptive hypertrophy of skeletal muscles is closely related to the recruitment of Ca^2+^ signaling satellite cells for repairing and regenerating skeletal muscle cells torn and damaged during exercise [83,87].

Kisspeptin activates GPR54 on the cytomembrane, which causes GPR54 and phospholipase C to combine with the G proteins of the Gαq/11 subfamily, eliciting the degradation of phospholipase C (PLC) in cells [88]. The degradation of PLC produces two types of second messengers: diacylglycerol (DAG) and trisphosphate (IP-3) [81]. IP-3 can increase the level of cellular Ca^2+^ [81,82]. Although these results were obtained from studies on the Kiss1/GPR54 system and cancer, given the existence of tissue interactions, we can speculate that Kiss1/GPR54 system is involved in the regulation of skeletal muscle Ca^2+^ concentration; this hypothesis needs to be confirmed by further studies.

In addition, increased kisspeptin signaling activates another second messenger, DAG, which facilitates the PKC pathway, and PKC activates MAPKs [89]. In mammals, MAPKs are involved in hormonal, neural, and cell division signaling. MAPKs interact with Ca^2+^ in the musculature and coordinate the regulation of skeletal muscle development [90]. Moreover, MAPKs phosphorylate ERK1/2. In myoblasts, ERK2 promotes myogenic progenitor proliferation by upregulating the expression of cyclin D1 [4]. In addition, the activity of MAPK p38 is induced during the differentiation of L8 myogenic cells, thereby promoting myogenesis [91,92]. The changes in Ca^2+^ signaling induced by *Kiss1* at the subcellular level provide important research ideas to investigate the link between *Kiss1* and skeletal muscle. Whether *Kiss1* can similarly promote changes in Ca^2+^ concentrations in skeletal muscle and thus affect the proliferation of skeletal muscle cells may be the next step in research.

### 4.2. Kiss1/GPR54 System in Mitochondrial Activity

There are two distinct populations of mitochondrial subpopulations in skeletal muscle. They are classified as intermyofibrillar mitochondria (MitoIMF) and subsarcolemmal mitochondria (MitoSS) according to the location of their existence [93]. During long-term aerobic exercise, the proportion of slow-twitch fiber has increased, which subsequently causes an increase in the activity and number of MitoIMF [93,94], but the mechanism is still unclear.

In the human melanoma cell, *Kiss1* inhibits the acetyl-CoA carboxylase phosphorylation by AMPK directly or upregulates PGC1α mRNA to activate the *AMPK* expression [95,96], which enhances mitochondrial β-oxidation and thus reverses the Warburg effect [97,98,99]. *Kiss1* can also directly promote mitochondrial biogenesis by regulating the expression of PGC1α [95]. Our previous study has shown that Kiss1 and GPR54 mRNAs can be detected in the skeletal muscles of C57Bl6 mice. The concentration of kisspeptin is stabilized at low-p mol levels in the plasma [65]. Given that the enhancement of mitochondrial activity in the skeletal muscle is reflected by the ability to oxidize fatty acids and substrates [100], which is consistent with the promotion of mitochondrial biogenesis by *Kiss1* in human melanoma cells, we speculate that the role of *Kiss1* in promoting mitochondrial β-oxidation may be present in the skeletal muscle.

**Figure 4:**
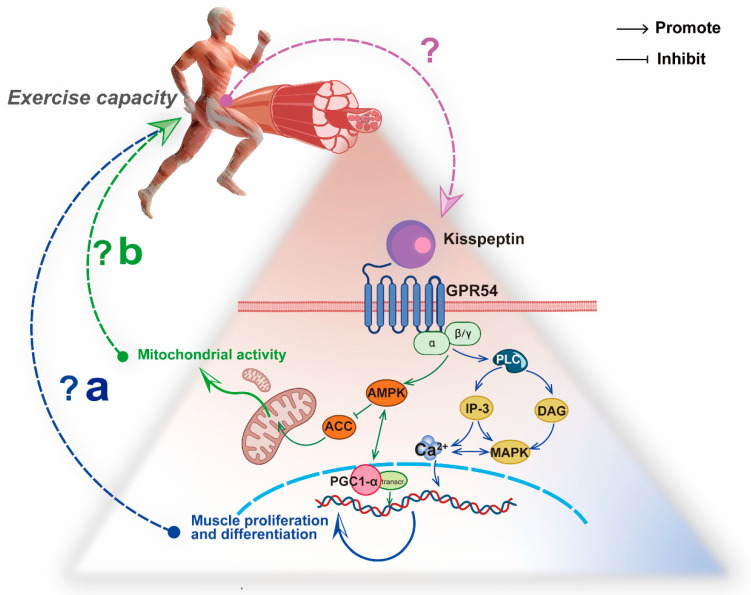
The hypothesis between the Kiss1/GPR54 system in skeletal muscle and exercise capacity. (**a**) Kisspeptin regulates Ca^2+^ concentration in skeletal muscle to promote proliferation and differentiation via the PLC-MAPK/IP-3 pathway. (**b**) Kisspeptin inhibits *ACC* in skeletal muscle to promote mitochondrial activity by APMK and PGC1α.

During high-intensity exercise, the phosphagen system provides approximately 50% of the ATP in the first 6 s, and the predominant ATP producer is the glycolysis system in the next 10 s [101]. Increased levels of Ca^2+^ and inorganic phosphate released from the sarcoplasmic reticulum lead to a high level of pyruvate production through glycogen breakdown [102]. Pyruvate can either be metabolized in the cytoplasm to produce lactate or enter the mitochondria for oxidation [102]. This process requires both the stability of Ca^2+^ levels and the adequacy of mitochondrial capacity [83,100]. Therefore, combined with the fact that *Kiss1* can affect Ca^2+^ signaling and mitochondrial β-oxidation in tumor biology, we propose that the Kiss1/GPR54 system in the skeletal muscle might have an implication for energy metabolism during exercise (Figure 4).

## 5. Conclusions

Energy metabolism is a complex process. As a multifunctional hormone, kisspeptin plays a role in the regulation of energy metabolism in the hypothalamus, liver, pancreas, and adipose tissue. Therefore, the Kiss1/GPR54 system provides a new perspective on energy systems. More studies should be carried out to explore the Kiss1/GPR54 system mechanism of action in different tissues.

## Data Availability

This review did not report any data.
